# Supplementary figures and images for: Biomedical Data Commons (BMDC) prioritizes B-lymphocyte non-coding genetic variants in Type 1 Diabetes
Source: PLoS Comput Biol. 2021 Sep 20;17(9):e1009382. doi: 10.1371/journal.pcbi.1009382 (PMC8483327; doi:10.1371/journal.pcbi.1009382)

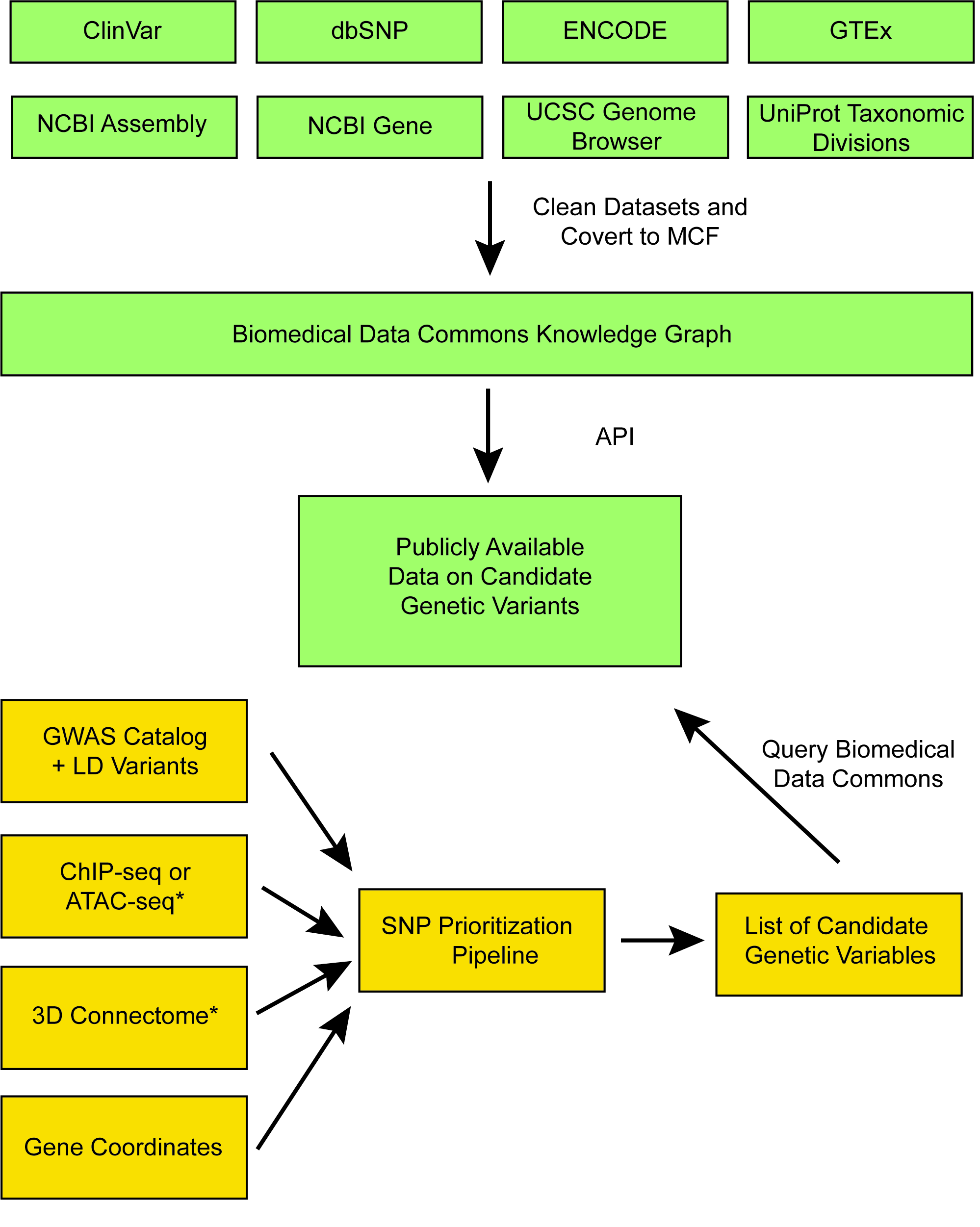

Supplement: S1 Fig — Biomedical Data Commons was built by converting publicly available datasets into MCFs, which were then ingested into the knowledge graph. This makes the data searchable using the Data Commons API. Private multidimensional ‘omics data was used as input into the SNP Prioritization Pipeline. This resulted in a list of candidate genetic variants, which were then used to generate queries to extract publicly available information on these variants from Biomedical Data Commons. Data and data processes relating to Biomedical Data Commons are in green and those related to the private data used in this study along with the developed SNP Prioritization Pipeline are in yellow. *denotes input data that is cell type-specific and needs to belong to the same cell type of interest. (TIF) [file pcbi.1009382.s001.tif]

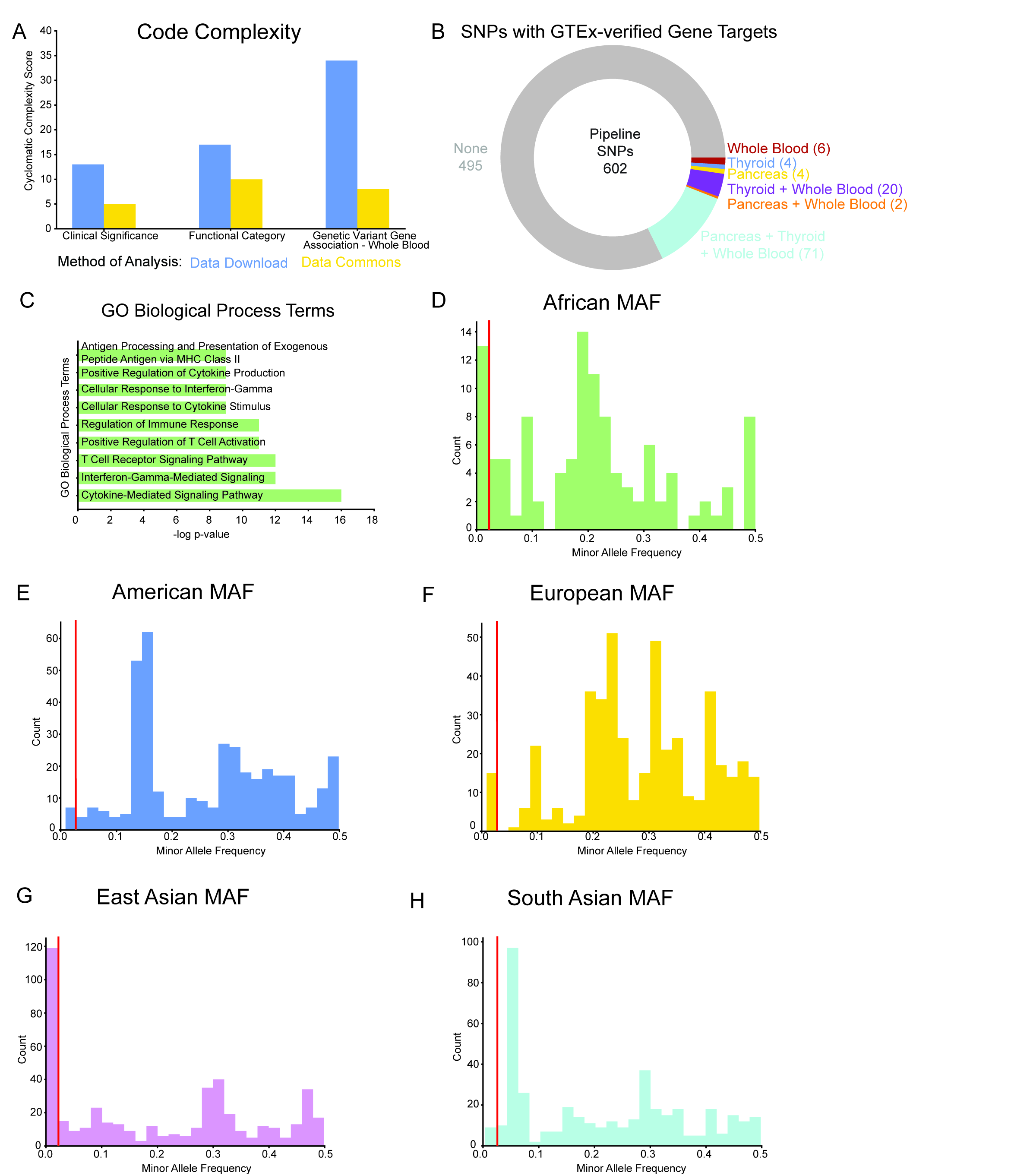

Supplement: S2 Fig — A: The code cyclomatic complexity score for the custom scripts used to identify the clinical significance, functional category, and significant gene association analyses using local data scientist approach involving data download (blue) or Data Commons (gold). B: Donut plot of genetic variants in which at least one gene target was verified by GTEx significant genetic variant—gene association in Whole Blood (burgandy), Thyroid (blue), Pancreas (gold) or a combination of tissues–Thyroid + Whole Blood (purple), Pancreas + Whole Blood (orange), or Pancreas + Thyroid + Whole Blood (turquoise). Genetic variants for which none of its gene targets were a GTEx significant genetic variant–gene association are in silver. C: Gene Ontology (GO) terms for genetic variants SNP pipeline identified gene targets. D-H: Minor allele frequency of pipeline genetic variants in specific subpopulations: African (D; green), Americans (E; blue), European (F; gold), East Asian (G; plum), and South Asian (H; turquoise). The red line is at minor allele frequency 0.02. (TIF) [file pcbi.1009382.s002.tif]

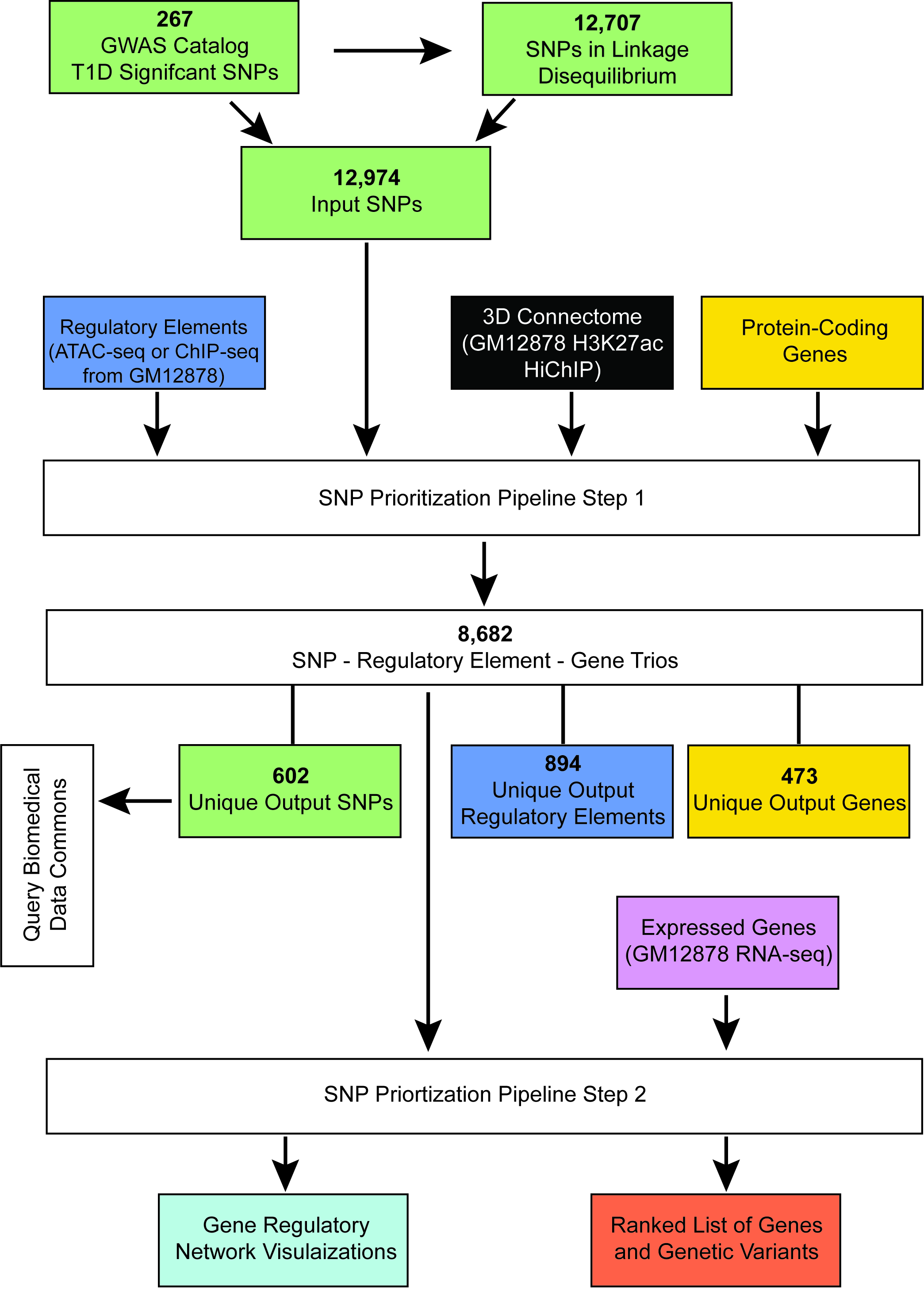

Supplement: S3 Fig — The original input list of genetic variants was generated by retrieving the Type 1 Diabetes significantly associated variants from GWAS Catalog (267) and then finding the genetic variants in linkage disequilibrium with those significant variants (12,707). Together these 12,974 variants were used as input into the SNP Prioritization Pipeline along with cell type-specific data on regulatory elements and 3D connectome as well as protein-coding genes genomic positions. The pipeline outputted associated genetic variants—regulatory elements—genes, which together formed trios. The number of unique genetic variants, regulatory elements, and genes that participate in these trio conformations in GM12878 cells using H3K27ac HiChIP input data are represented. The gene list from step 1 of the SNP Prioritization Pipeline is then used as input for Biomedical Data Commons queries. In addition, the trios generated in step 1 are used as input into step 2 of the SNP Prioritization Pipeline along with cell type-specific gene expression data. The output is visualizations of gene regulatory networks and a ranked list of genetic variants and genes. Input and output data at each step of the pipeline is color coded by type of data: genetic variants (green), regulatory elements (blue), genes (gold), 3D connectome (black), gene expression data (magenta), gene regulatory networks (turquoise), and ranked list of genetic variants and genes (orange). (TIF) [file pcbi.1009382.s003.tif]

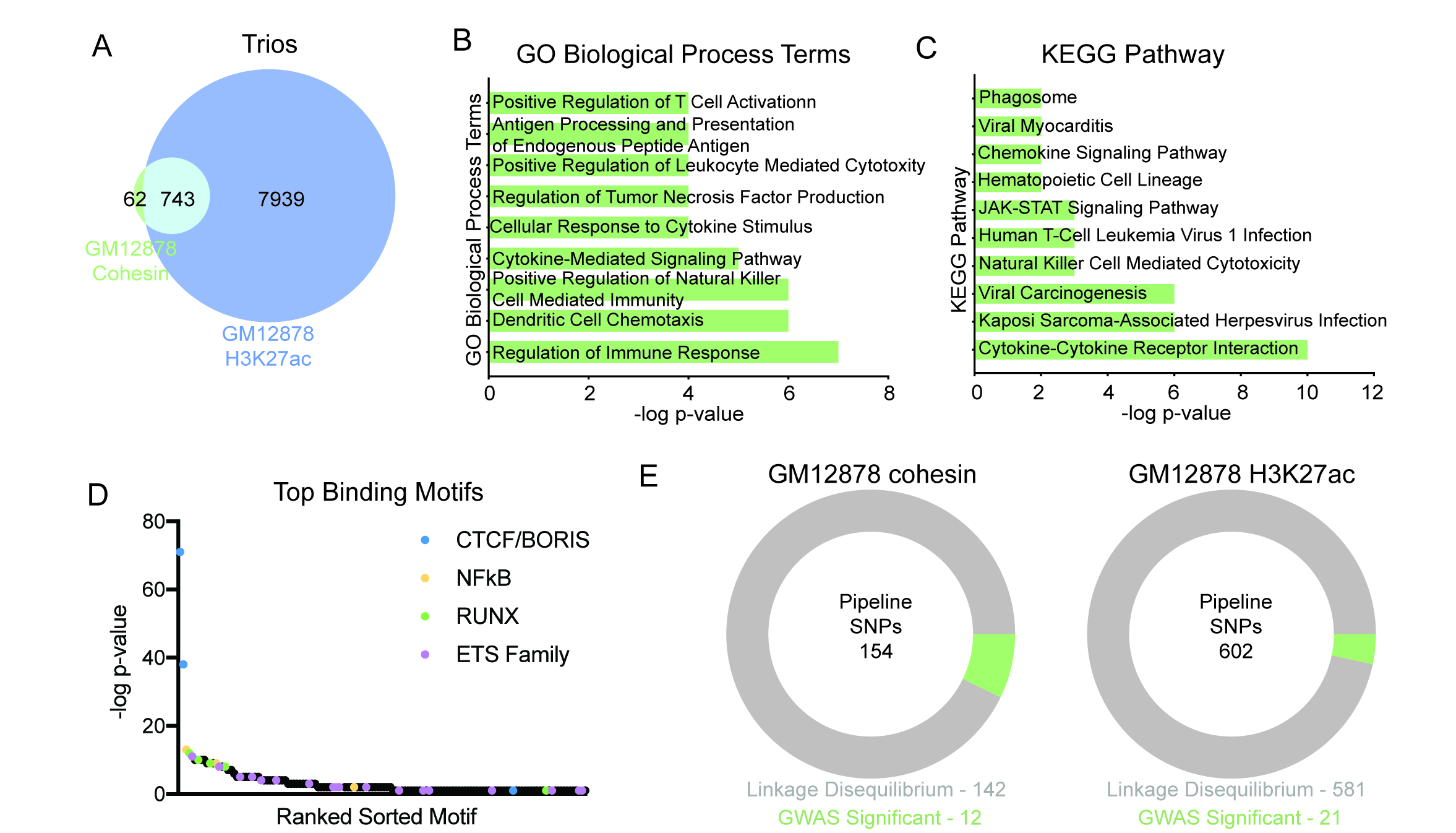

Supplement: S4 Fig — A: Venn diagram of trios generated with cohesin HiChIP (green) vs H3K27ac HiChIP (blue) with trios common to both datasets in green. B: Gene Ontology (GO) terms for genetic variants SNP pipeline identified gene targets with cohesin HiChIP as input. C: Kegg pathways for genetic variants SNP pipeline identified gene targets with cohesin HiChIP as input. D: Scatter plot of the top binding motifs of pipeline identified regulatory elements with cohesin HiChIP as input. E: Donut plot of the number of pipeline genetic variants that are significant in a Type 1 Diabetes GWAS study (green) or are in linkage disequilibrium with a significant genetic variant (silver) with cohesin HiChIP (left panel) or H3K27ac HiChIP (right panel) as input. (TIF) [file pcbi.1009382.s004.tif]

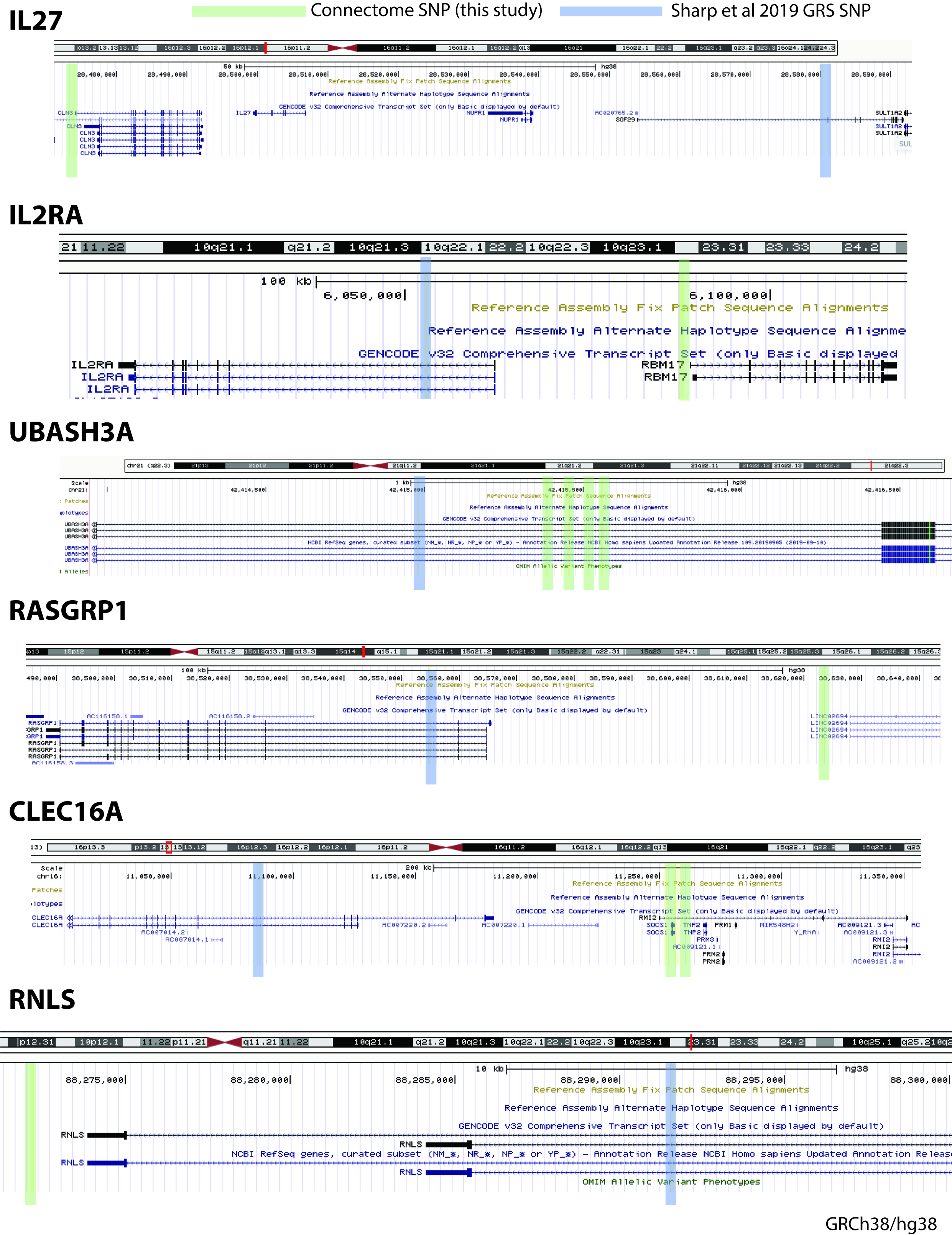

Supplement: S5 Fig — GRS2 study (2019). In addition to identical calls as the Sharp et al. GRS2 study, the SNP pipeline identifies variants (green) nearby GRS2 variants (blue) that have the same gene targets (2019). These variants in close physical proximity include those in IL27, IL2RA, UBASH3A<RASGRP1, CLEC16A, and RNLS loci. The difference between these two studies is that this connectome study (blue) restricts SNPs to regulatory elements and uses cell-type specific biological data as input whereas the GRS2 study (green) performs a statistical analysis of UK BioBank records to identify variants. (TIF) [file pcbi.1009382.s005.tif]

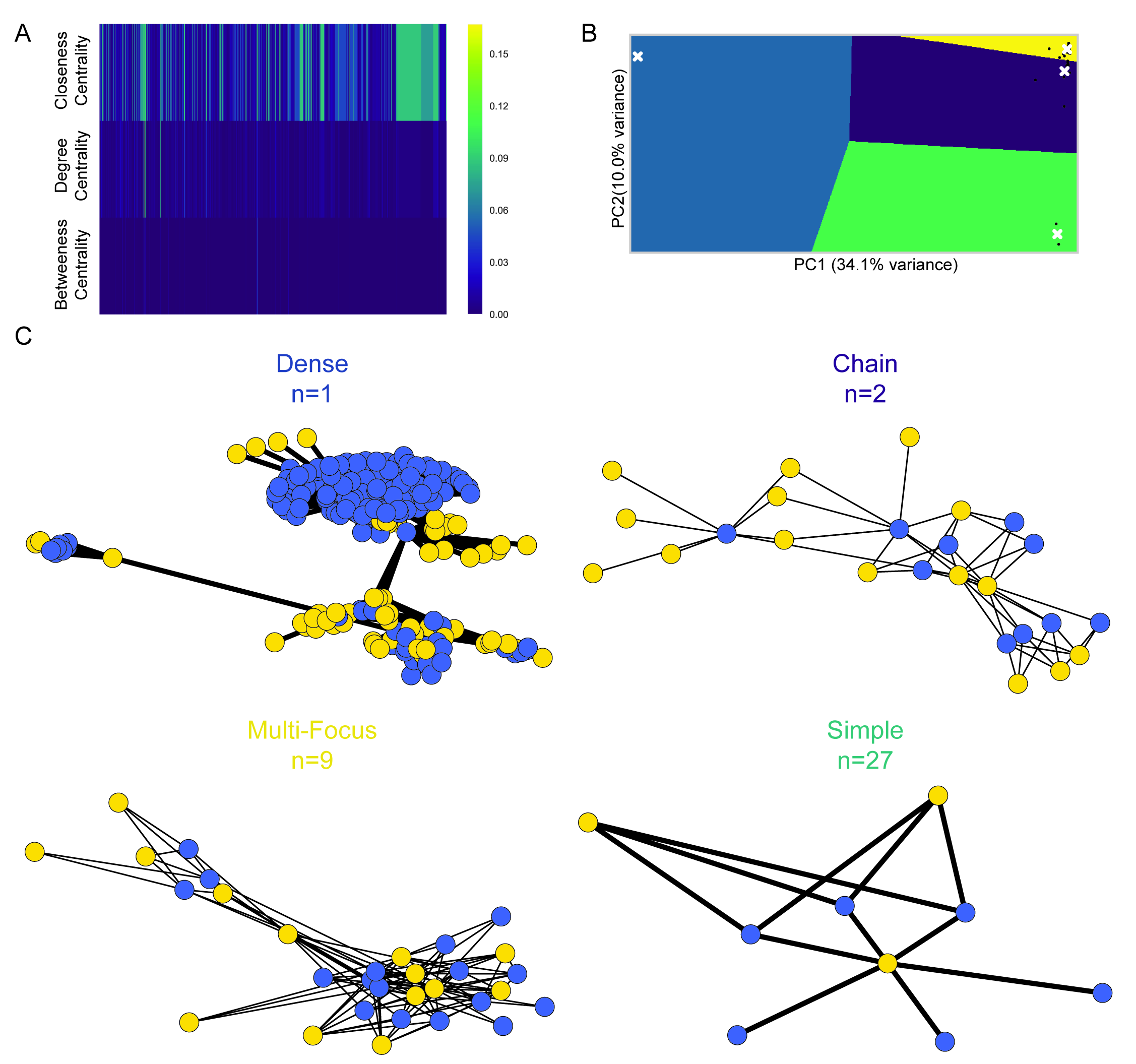

Supplement: S6 Fig — A: Heatmap of the closeness, degree, and betweenness centrality of the nodes in the genetic variant–gene regulatory networks. B: PCA of the matrix representation of the components of the genetic variant–gene regulatory network. K-means clustering (k = 4) was performed on the PCA and the boundaries are represented by the colors. Each black dot is a component and the white X’s mark the centroid of a cluster. C: Components of the genetic variant (blue)–gene (gold) network that represent each cluster of components. (TIF) [file pcbi.1009382.s006.tif]

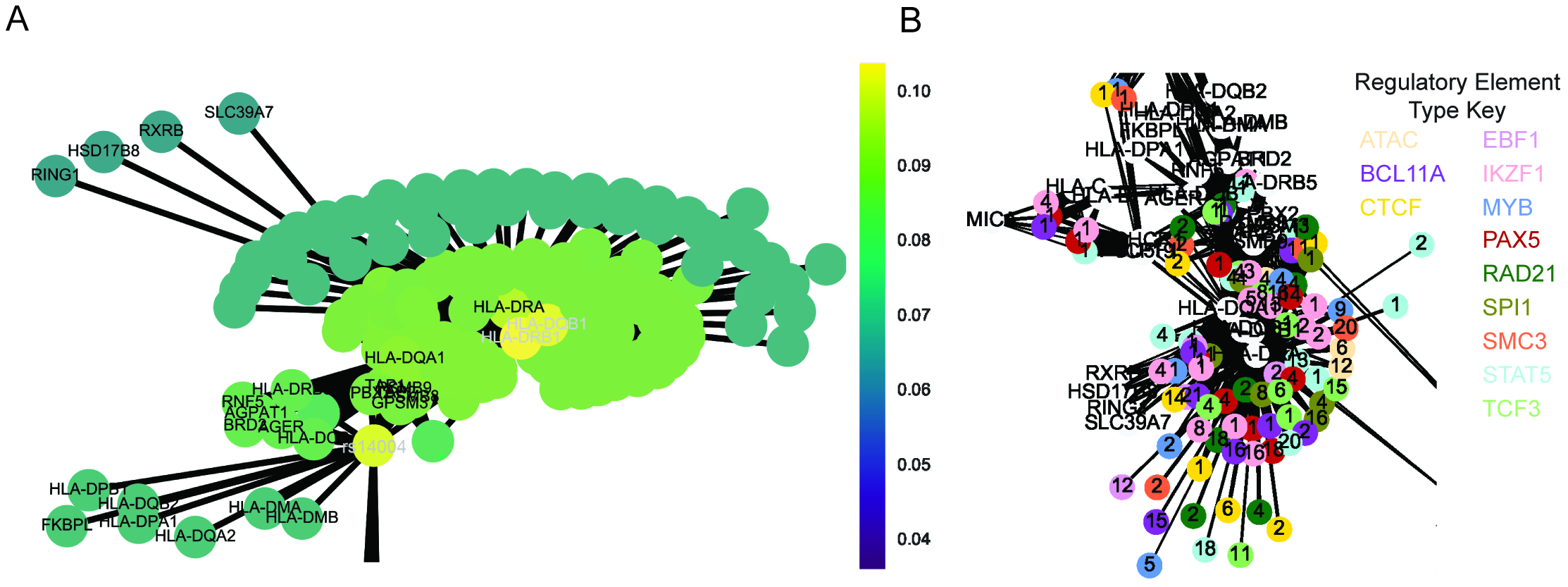

Supplement: S7 Fig — A: Zoomed in bipartite graph of the 1 Mbp HLA component containing top candidates HLA-DQB1, HLA-DRB1, and rs14004 with gene and genetic variants as nodes and chromatin connections as edges. Node color indicates closeness centrality score with gold being most connected and purple being least connected nodes in the graph. Gene nodes are labeled, and genetic variant nodes are unlabeled. B: Zoomed in bipartite graph of the 1 Mbp HLA component HLA component containing top candidates HLA-DQB1, HLA-DRB1, and rs14004 with gene and regulatory elements as nodes and chromatin connects as edges. Gene nodes are labeled and white. Regulatory element nodes are colored by type and labeled by the number of unique genetic variants contained in the regulatory element. The width of edges indicates connectivity strength as indicated by the number of unique HiChIP reads. (TIF) [file pcbi.1009382.s007.tif]

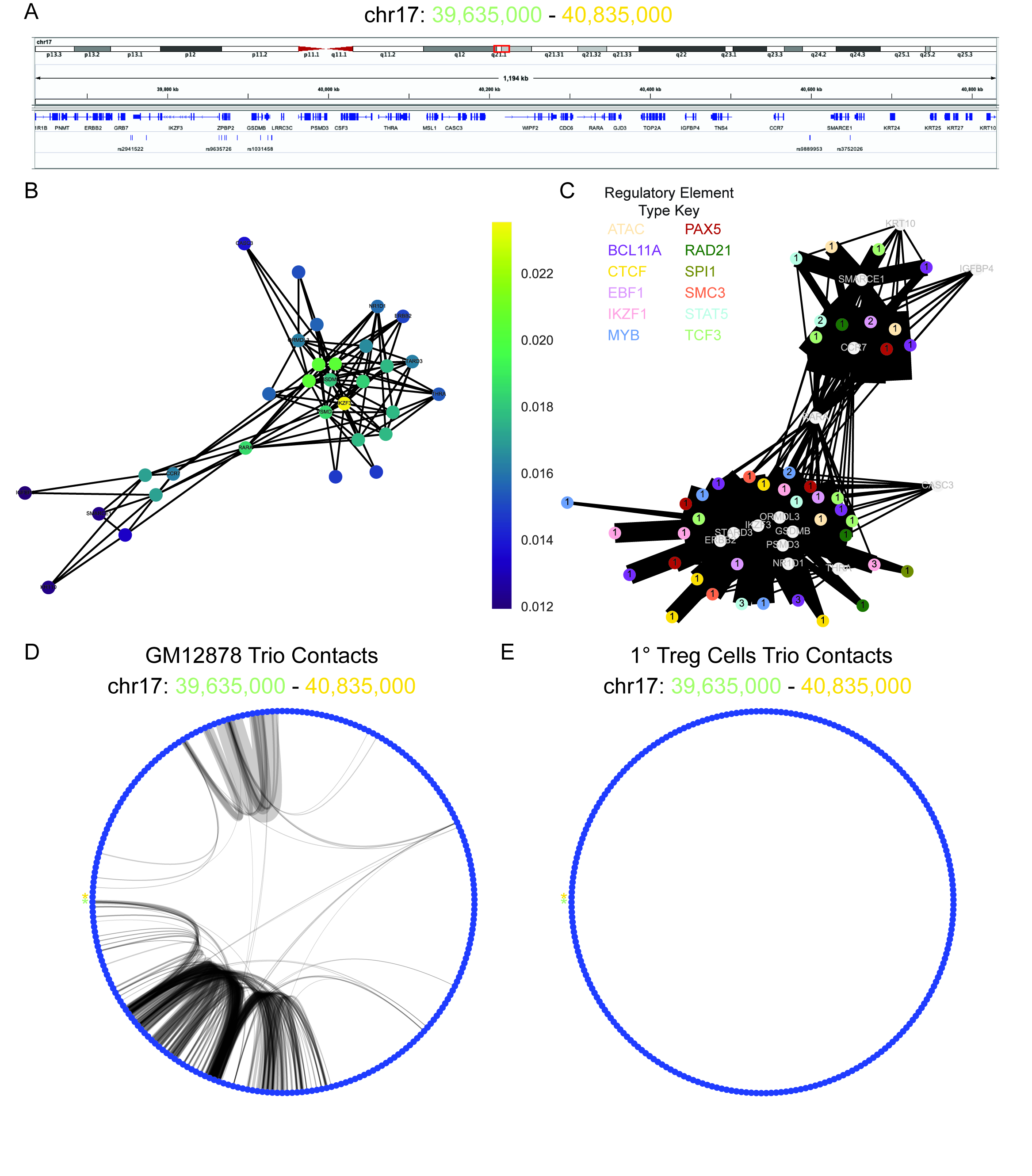

Supplement: S8 Fig — A: Visualization of the IKZF3 component of interconnected pipeline genetic variant–regulatory element–gene trios (chr17: 39,635,000–40,835,000). B: Bipartite graph of the IKZF3 component with gene and genetic variants as nodes and chromatin connections as edges. Node color indicates closeness centrality score with gold being most connected and purple being least connected nodes in the graph. Gene nodes are labeled, and genetic variant nodes are unlabeled. C: Bipartite graph of IKZF3 component with gene and regulatory elements as nodes and chromatin connects as edges. Gene nodes are labeled and white. Regulatory element nodes are colored by type and labeled by the number of unique genetic variants contained in the regulatory element. The width of edges indicates connectivity strength as indicated by the number of unique HiChIP reads. D: Circos plot of the chromatin connectivity at 5 kb resolution in the IKZF3 locus. The nodes are sections of the genome and the edges are the chromatin connectivity with the width indicating connectivity strength. An asterisk labels the starting (chr17: 39,635,000; green) and terminating (chr17: 40,835,000; gold) nodes of the plot. GM12878 (left panel) and Treg (right panel) pipeline trio contacts are visualized. (TIF) [file pcbi.1009382.s008.tif]

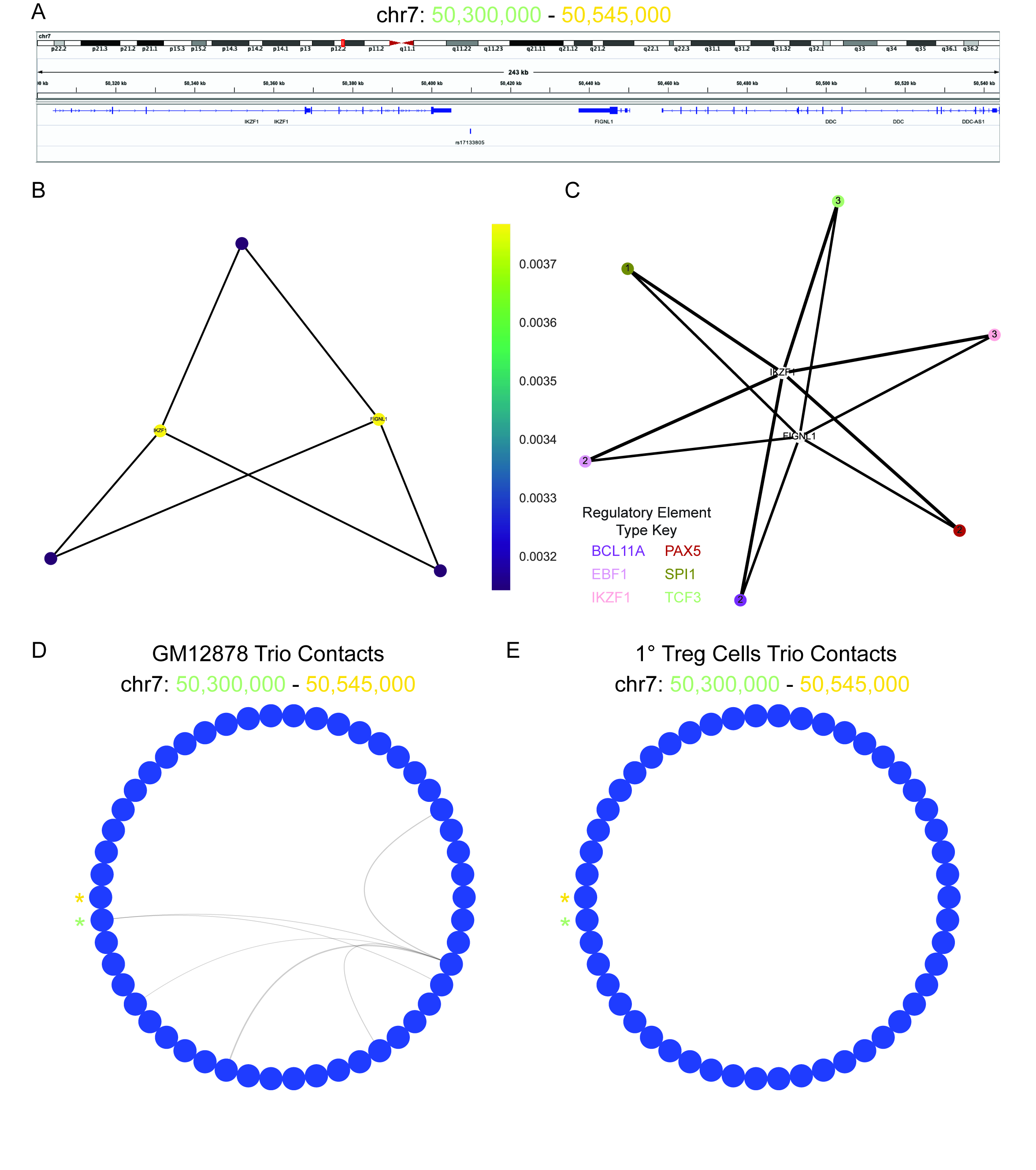

Supplement: S9 Fig — A: Visualization of the IKZF1 component of interconnected pipeline genetic variant–regulatory element–gene trios (chr7: 50,300,000–50,545,000). B: Bipartite graph of the IKZF1 component with gene and genetic variants as nodes and chromatin connections as edges. Node color indicates closeness centrality score with gold being most connected and purple being least connected nodes in the graph. Gene nodes are labeled, and genetic variant nodes are unlabeled. C: Bipartite graph of IKZF1 component with gene and regulatory elements as nodes and chromatin connects as edges. Gene nodes are labeled and white. Regulatory element nodes are colored by type and labeled by the number of unique genetic variants contained in the regulatory element. The width of edges indicates connectivity strength as indicated by the number of unique HiChIP reads. D: Circos plot of the chromatin connectivity at 5 kb resolution in the IKZF1 locus. The nodes are sections of the genome and the edges are the chromatin connectivity with the width indicating connectivity strength. An asterisk labels the starting (chr7: 50,300,000; green) and terminating (chr7: 50,545,000; gold) nodes of the plot. GM12878 (left panel) and Treg (right panel) pipeline trio contacts are visualized. (TIF) [file pcbi.1009382.s009.tif]
