# Supplementary material for: Biomedical Data Commons (BMDC) prioritizes B-lymphocyte non-coding genetic variants in Type 1 Diabetes
Source: PLoS Comput Biol. 2021 Sep 20;17(9):e1009382. doi: 10.1371/journal.pcbi.1009382 (PMC8483327; doi:10.1371/journal.pcbi.1009382)
Supplement: Attachment — Submitted filename: Response_to_Reviewers_PLoS_Computational_Biology.docx [file pcbi.1009382.s018.docx]

Reviewer's Responses to Questions

**Comments to the Authors:
Please note here if the review is uploaded as an attachment.**

We have uploaded our responses to the reviews as an attachment.

Reviewer #1: The authors have written a nice manuscript describing a new interface to understand clinically relevant high dimensional data and an example therein. The example is a vignette describing the use of orthogonal genetic and epigenetic datasets to elucidate the potential epigenetic consequences of common germline SNPs in type I diabetes. They identify a majority occur in regulatory regions identified by ATAC-Seq and two HiCHIP datasets in B cells (most of which are confirmed in two T cell datasets). The data and methodology appear interesting. I would say that the manuscript itself is dense. It is difficult to follow because terminology is almost always the same even if the assays are different. I would highly encourage having supplementary figures for workflows and files for methods to describe how things were done. I describe a few examples below but there are more. I would encourage the authors to be proactive about this and not simply address these few examples i have listed here.

Major comments:
1. Though the authors show vignettes describing how there are some “B-cell specific” variants, i.e. those that occur in MHC class II loci, it is not clear if or why B-cell datasets are preferable to T cell datasets. IS the B-cell dataset preferable (meaning it explains a higher number of variants overall?)

We thank the reviewer for their comment on the need to clarify why we selected focusing on B-lymphocytes rather than T-lymphocytes as the main focus for this paper. We have included additional information in the Intro and Discussion clarifying the reasoning for this selection. We selected B-lymphocytes as the focus in order to demonstrate how BMDC and the SNP Prioritization Pipeline can provide biomedical insight into the role of genetic variance in the largely overlooked cell-type. We find the existence of B cell specific disease associated regulatory elements controlling the same genes as in T cells, implicating gene dysregulation in B cells in T1D pathogenesis.

We selected immortal cell line GM12878 in particular due to the extensive publicly available data on it including ChIP-seq of eleven transcription factors that are critical for B-lymphocyte development and cell identity. This resulted in the identification of 602 pipeline-identified variants of interest in B-lymphocytes. We compared this to the pipeline results using 1° Naive T-cells, 1° Th17 helper cells, and 1° Treg Cells in Figure 3E. The results using the T-cell data was much more limited because there were no transcription factor ChIP-seq datasets for any of the 1° T-cell datasets. Only ATAC-seq datasets (open chromatin regions) were publicly available. This resulted in hundreds of less variants being identified by the pipeline in these T-cells. In contrast, the GM12878 with the multitude of publicly available datasets provided a more complete picture of the epigenetic landscape of the cell. This provided a better platform to showcase the usefulness of BMDC and SNP Prioritization Pipeline. Furthermore, it increases the novelty of the results reported in this paper by expanding understanding of how genetic variance may impact known loci like MHC class II and IL2RA, as well as less studied loci in B-lymphocytes. This provides a greater benefit to the field than focusing on the well curated T-lymphocyte.

2. The manuscript is too dense. It is difficult to follow what was exactly done. I’ll provide multiple examples here but the examples affect actually every aspect of the manuscript.

The reviewer brings up an important issue regarding the clarity of the manuscript. To address this, we have taken care to reduce the number of acronyms used in the manuscript as well as expand naming and description of specific analytic methods used in the manuscript. We took care to reword particularly confusing sections of the text, especially those pointed out by the reviewers, to aid in readability of the manuscripts. We have also expanded the description of the results and methods section to provide more detail and clarity of how the analyses were conducted. In addition, schematics were added as Figure 2, Supplementary Figure 1, and Supplementary Figure 3 to aid in understanding of the data and data processes deployed in this study. We also added Supplementary Table 6-8 to increase clarity of the unique schema developed for building the Biomedical Data Commons. Finally, we added links to additional documentation on Biomedical Data Commons and the data analyses to provide the reader with more information on the data and analyses deployed in this study than what is able to reasonably fit within the manuscript itself.

A) PCA plots in supplementary figure 4. What are each dots? They represent a node? How exactly were the nodes identified? PCA’s were generated in A and B seem similar except they use different legends? The legend for D looks similar to A and B but somehow succeeds C which is about centrality. The k-means clustering appears to be done after PCA, but the PC1 and PC2 change by this analysis? What was done here?

We thank the reviewer for pointing out that the original Supplementary Figure 4 was unclear. Please note that in this draft it is now Supplementary Figure 6. An advantage of representing data graphically is that in recent years there have been major developments in Representation Learning. These methods enable a graph to be represented as mathematical vectors, which enables researchers to perform Machine Learning to extract insights. Here, we deployed it to cluster similar gene regulatory network structures together by performing k-means clustering on the PCA on the gene regulatory networks. To make it clearer the Results subsection “There are four clusters of gene regulatory network structures” and the Methods subsection “Representation learning of the genetic variant – gene regulatory networks” were reworded and expanded upon to include additional information and motivation for this analysis

We removed the original Supplementary Figure 4A-B from Supplementary Figure 6 because they were unnecessarily distracting and not important for the conclusions made from the representation learning on the networks. They were initially included to provide more information on the similarities of nodes (genetic variants or genes) in the bipartite graph across all graph components. It was supplied as an additional analysis to give the reader more context of the data. However, we see how this distracted from the narrative on the analysis of the gene regulatory network structure and therefore removed them from the new Supplementary Figure 6.

B) identifying the nodes. Presumably in supplementary figure 4E, the dense cluster plot indicates the reason why the authors focused on MHC class II. However, how exactly were IL2RA, IKZF3 chosen? The legends seems to indicate they were the top candidates chosen by different analyses but which analyses? Do they reflect different nodes in the cluster plots? How do these graphs connect to one another?

The purpose of the vignettes diving into select loci of interest was to validate that our pipeline is finding and prioritizing biologically relevant genetic variants and gene regulatory networks. We therefore decided to present 4 loci of varying complexity and belonging to different structural network clusters identified in this study. This demonstrates to the reader how the output of the SNP Prioritization Pipeline can be used at different loci to extract biological insight that can be used to inform validation experiments. A main reason why these loci were selected is because there is existing literature on the role of these loci in Type 1 Diabetes. The purpose of these vignettes is to highlight how what we found is congruent with the literature and adds to the conversation concerning the potential mechanism by which dysregulation contributes to Type 1 Diabetes disease phenotype. This provides confidence that the output of this method is providing candidate regions that are promising for biological validation. We added salient details to the selection of each loci in the results section and added the Methods subsection “Selection of loci of interest” to share this reasoning.

C) figure 2J. What are the output SNPs? The results describe utilizing SNPs in databases and then prioritizing/categorizing them based on their presence in various databases. I presume these are the input SNPs? What are the output SNPs? Are these the SNPs that are found by

The input SNPs refers to the original candidate list of 12,974 genetic variants that were reported significantly associated with T1D in the GWAS catalog or are in linkage disequilibrium. The Output SNPs refers to the 602 genetic variants that were identified as candidates by the pipeline. To increase clarity of this specific instance these definitions were added to the figure legend of Figure 2J. These definitions were also added to the Results subsection “Output candidate variants are common and not evolutionarily conserved”. In addition, to aid in understanding of the data and data analyses workflow schematics representing the data analysis process were added in Figure 2, Supplementary Figure 1, and Supplementary Figure 3. Specifically, Supplementary Figure 3 shows the workflow of prioritizing variants specifically in GM12878 and the quantified input and output data at each step of the process.

D) Figure 1C. Genetic variant gene association. Presumably this is the regulatory region of the trio described in the text?

Figure 1C was provided to introduce the concept of a user being only interested in a subset of the graph being able to extract the desired information that they want at scale. An expanded description of Figure 1C along with the user motivation is included in the Result subsection “Biomedical Data Commons integrates multiple data types into a single graph” to clarify this point. The figure legend for Figure 1C was also reworded and expanded to increase clarity.

E) I can add more vignettes like this. I think the paper seems well done, but the writing is 1) so dense 2) leaves out salient details 3) uses almost identical terminology to describe different algorithms that lead to different results. I would highly recommend that the reviewers have someone who was not involved in the project read the paper and try to elucidate how the data was generated and represented. If the word limits preclude a sufficient description of the methods, i would include them in a supplementary document.
We thank the reviewer for highlighting the need to increase readability and provide more detail for the analyses. We addressed this general comment by rewording throughout the paper to increase clarity. We also added salient details to the Results section to aid in the understanding of the purpose of analyses, which aids narrative flow. In addition, we have greatly expanded the methods section to include much more detailed information in the building of Biomedical Data Commons and the SNP Prioritization Pipeline. We added more details about the workflow to make it clear which data was used where and what did the analyses generate. Finally, we added 3 additional Supplementary Figures and 4 additional Supplementary Tables to provide additional information and clarity to the workflow and the analyses. In particular, we would like to highlight Supplementary Figures 1 and 3, which are schematics of the workflow of the paper and the SNP Prioritization Pipeline respectively. We hope that this has increased the readability and interpretability of the paper.

Minor comments:
1. Comments such as B-cell specific may be misleading. MHC class II is canonically expressed by professional antigen presenting cells such as B cells. T cells are not traditionally considered ones. However, it may be possible that this observation is not shared with T cells but may be shared with other cell types such as dendritic cells.

The reviewer brings up a good point on the caveats of the study presented here. In response we added a paragraph on the limitations of the study to the discussion and how they could be addressed in future studies. We made sure to include the point brought up by the reviewer that we did not look at dendritic cells or other cell types like B-islet cells that are known to be involved in Type 1 Diabetes. We propose that these cell types be examined using the method described in this study in future research.

2. Scattered typos in the headers.

We thank Reviewer 1 for pointing out that we had distracting typos. We took care to correct these.

Reviewer #2: There are some minor points which need to be more expanded in the manuscript:
Methods:
Please explain the schema of your developed data base more, such as the relationship between the integrated databases.

The reviewer requested more information on how the schema of the database was developed. In response, we have added a new Methods subsection “Biomedical Data Commons schema development”. This defines the data model for Biomedical Data Commons, expands that it is built upon schema.org and expands on how new schema unique to Biomedical Data Commons is generated. We have also added Supplementary Tables 6-8 that define the unique Biomedical Data Commons schema as of 4/9/21. In addition, the most up to date version of the schema can be viewed on the Data Commons browser (datacommons.org) or in the schema section of the datacommonsorg git repository as previously noted in the Methods subsection on “Data and code Availability”.

In a part of your method please explain the data cleansing you done for the integration of data, how did you handle data type or data structure mismatch?

The reviewer requested additional information on the data cleaning and integration into Biomedical Data Commons. We have therefore added the new Methods subsection “Ingesting data into Biomedical Data Commons”. This documents how the data is parsed and formatted prior to ingestion. It also describes how data across multiple datasets are joined and mapped to common entities in the knowledge graph through careful generation of a Data Commons ID (dcid). The specifics on the data cleaning and mapping to dcid for each import is too lengthy to include in the paper itself. Instead, we have made it available on our git repository here (<https://github.com/datacommonsorg/data/tree/master/scripts/biomedical>). For each dataset we provide documentation on the dataset and how it was imported into Biomedical Data Commons including all scripts used in data cleaning and conversion to MCF.

Please draw a comprehensive sketch of your developed ontology, it is very hard to follow the whole idea through the method part.

In addition to the previously provided Supplementary Table 1, which reports the Biomedical Data Commons’ entities, the number of nodes of each type in the graph and the original data sources, and Supplementary Table 2, which reports the number of edges of each general category, we have added Supplementary Table 6-8. Together these define all of the entities, properties, and enumerations that are unique to Biomedical Data Commons included in this study. Furthermore, we added the Methods subsection “Biomedical Data Commons schema development”, which defines how ontology is generated. As previously, noted Data Commons is built upon schema.org and only extends the schema as needed to accommodate new datasets. The ontology of schema.org is heavily documented and can be found here (<https://schema.org/docs/documents.html>). Additional documentation on the Data Commons Data Model can be found on the Data Commons browser (<https://docs.datacommons.org/data_model.html>).

Discussion:
Please compare the advantages of you study in comparison to other studies.
What were the limitations of your study and how did you handle them?

We added a paragraph to the discussion comparing Biomedical Data Commons to two other biomedical knowledge graph efforts: The Biomedical Data Translator Consortium and the University of California, San Francisco SPOKE graph. We also added two paragraphs discussing the limitations of this study, how we addressed them, and future research directions.

Reviewer #3: This work aims at showcasing Biomedical Data Commons that here is used to query a knowledge graph of genomic, epigenomic, and transcriptomic data from seven databases. The field of application is type 1 diabetes – but the choice of analysis is on the less understood role of B lymphocytes. The basic concept is that of calling regulatory elements, mapping variants in a specific B cell line (GM12878) and assigning the regulated gene by H3K27ac HiChIP. There is then a number of statistical genetic assumptions and analyses that start with the assessment of variants that compose a genetic risk score of T1D based on GWAS. Individually, the various steps are conducted correctly.

There are a number of issues that diminish the impact of this work. They can be grouped in: biological rational, study design and statistical support, study interpretation.

1. Biological rational: there are several possibilities to benchmark BMDC: an area of advanced knowledge, or an unknown area of knowledge in a disease. T1D has a well-defined basis in T cell immunity and it would have been interesting and important to understand the meaning of the polygenic basis of disease in those cells. However, the authors chose the more complex path of B cell biology. The workhorse of the work is an immortalize B cell line that provides the data to support the analysis. The biological assumption is that this cell in some form, reflects the biology of a primary B cell in a patient. It is clear that that data currently available is what it is – but the decision to use immortalized B cells makes the assessment of result complex.
We thank the reviewer for pointing out that we needed to further clarify our selection of B-lymphocytes as the main subject for the paper. As mentioned previously, we selected B-lymphocytes as the focus in order to demonstrate how BMDC and the SNP Prioritization Pipeline can provide biomedical insight into the role of genetic variance in the largely overlooked cell-type. We find the existence of B cell specific disease associated regulatory elements controlling the same genes as in T cells, implicating gene dysregulation in B-lymphocytes in Type 1 Diabetes pathogenesis.

To address this we added more information to the Introduction and Discussion section clarifying the reasoning behind selecting B-lymphocytes. We also acknowledged the limitation of using the immortalized cell line in the Discussion. One of the reasons for selecting the GM12878 is that it is heavily characterized and has a wealth of publicly available datasets including eleven ChIP-seq datasets of important B-lymphocyte transcription factors. None of the three 1° T-cells had data on any of the transcription factors, which limited their interpretation and ability to showcase the method. Furthermore, as we address in the Introduction and Discussion sections, the B-lymphocyte contribution to Type 1 Diabetes disease manifestation and progression is less understood in contrast to the well-studied T-lymphocytes. We therefore used vignettes demonstrating the SNP Prioritization Pipeline and using BMDC in B-lymphocytes to increase the contribution value of this study to the field.

2. Study design and statistical support. It is really difficult to follow what has been done with variant selection. The manuscript refers to a list of 267 previously published significant T1D GWAS variants and an additional 12,707 variants in linkage disequilibrium. It also refers to the 67 variants in the genetic risk score. Then, there are 602 unique genetic variants participating in 8,682 unique genetic variant - regulatory element - gene trios. It is assumed that here, “variants” refer to the unique variants of the cell line and not to population variants. From this point on, there are a number of comments on prioritization that lacks a clear definition of the approach, the materials and tools to prioritize. There are a number of statements that more directly refer to overlaps/enrichment between population variants and lymphocyte variants – with unclear statistical statements. What is the basis for an statement of “strong connection”, etc.
There are also confusing statements such as:
Lines 219 to 226: “Genetic variants in non-coding regulatory elements - open chromatin regions or transcription factor binding sites - in GM12878 B-lymphocyte cells were associated with gene targets using three-dimensional chromatin conformation data defined by H3K27ac HiChIP……..The vast majority of these trio variants are of unknown clinical significance and they are enriched for non-coding regions compared to the input genetic variants.” - how can there be other than “enrichment” in non-coding regions: the input genetic variants are defined as being in the non-coding region.

To increase the ease of understanding of the study design we added Figure 2, which visualizes the data used as input for the first step of the pipeline and the output generated. We also added Supplementary Figure 3, which visualizes the workflow for the analyses done in GM12878 cells, the output of which was the main focus of this paper. This included how the original list of Type 1 Diabetes genetic variants was generated prior to input into the pipeline. It also includes the numbers for the output from the first step of the pipeline for the B-lymphocyte cells as this was the data for which the vast majority of the subsequent analyses of this paper focuses. In addition, the wording mentioned by the reviewer has been revised to clarify the results in Results subsection “SNP pipeline prioritizes non-coding and tissue-specific variants”. As the reviewer rightfully pointed out based on the pipeline's design we would expect that it would identify non-coding genetic variants as candidates. However, we still verified this expectation and provided more insight into the genomic location of the genetic variants that the pipeline selects in Figure 1C.

Furthermore, additional information was added to the Methods section. In subsection “Generating Type 1 Diabetes genetic variant list” the following sentence was added “This final generated Type 1 Diabetes-associated genetic variants list was used as input into the SNP Prioritization Pipeline in all analyses, regardless of cell type.” In addition we extensively expanded Methods subsection  “Pipeline for prioritizing non-coding genetic variants”  to provide more information describing the pipeline we developed. We made sure to specify the input and output data uat each step. Finally, additional information was added to subsection “Pipeline deployment for cell-type specific analysis”. Together this should make clear in our methods that 1) the input Type 1 Diabetes-associated genetic variant input list used in this study is the same regardless of the cell type of interest 2) how the data is processed in the SNP Prioritization Pipeline, 3) that the pipeline uses cell type-specific information as input for the regulatory elements and three-dimensional chromatin conformation only, and 4) we ran the pipeline several times in this study with different cell type-specific input data in order to address several different questions.

Another confusing statement is:
“On top of this platform, we created a pipeline that prioritizes genetic variants, which are primarily of unknown clinical significance and in the non-coding genome. These variants are specific to B-lymphocytes but are associated with genes previously implicated in Type-1 Diabetes, suggesting they effect cell type-specific gene regulation.” – what is understood for variants specific to B-lymphocytes: variants are either specific to a host, or to a B cells line (via transformation or oncogenesis” – but there are no universal variants to B cells.
We appreciate that the reviewer pointed out a section for which our wording was particularly unclear and we reworded this section to increase clarity. In addition, we provided schematics of the data and data processes used for data analyses reported here in Figure 2, Supplementary Figure 1, and Supplementary Figure 3. We would also like to point the reviewer to the pre-existing Method subsection “Generating input Type 1 Diabetes genetic variant list” for how the original Type 1 Diabetes genetic variant input list was generated. The variant input list is not cell type-specific information. To make it clearer what type of data used that was cell type-specific additional description information was added to the results section. We also expanded the Methods subsections “Pipeline for prioritizing non-coding genetic variants” and “Pipeline deployment for cell type-specific analysis” to include more detail about the data and data processes deployed in this study and which data was cell type-specific.

We would also like to take this opportunity to kindly point out that the definition of genetic variant used in this study is the standard definition of genetic variants - defined positions on the genome that have one or more alternate forms. The DNA with these variants are present in every cell type, but do not necessarily affect gene expression in any given cell type. The goal in this study is to develop a method that identifies genetic variants that are likely to play a role in a given disease by affecting gene expression in a specific cell type. In this study we explore the role of genetic variants in Type 1 Diabetes in B-lymphocytes as an example use case. We are not using the term genetic variant to refer to B-lymphocyte haplotypes at any point in the manuscript.

Down in the manuscript, there are many traditional pathway and clustering operations, assessment of GTEx etc, with a lack of clarity of what goes into what analysis and for which biological question. Of the 7 data sources, what was the exact use and contribution of each to the graph and the downstream analysis?
Perhaps the study would benefit from a clear figure that indicates what was done with what data and why.

The reviewer brings up an important point that a lot of data was used in this study from multiple different sources and it’s important to clarify what was used for which analyses. We therefore added Supplementary Figure 1, which displays the overview of the data and data processes conducted in relation to Biomedical Data Commons. It defines the data that was ingested into Biomedical Data Commons from publicly available databases. It also defines how the list of candidate genetic variants was generated from private (lab-generated) multidimensional ‘omics data that was then used to form queries to extract more publicly information on these variants from the Biomedical Data Commons knowledge graph using the Data Commons API. To make it clear how Biomedical Data Commons was generated this methods subsection was greatly expanded upon and the information is now represented in three new methods subsections: “Biomedical Data Commons schema development”, “Ingesting data into Biomedical Data Commons”, and “Biomedical Data Commons knowledge graph data storage and access”.

Furthermore, the providence (data source) for any information retrieved from Biomedical Data Commons is noted along with the analysis performed in the Results section. We also noted that this information was retrieved from Biomedical Data Commons using the Data Commons. Finally, I would also like to refer the reviewer to the Methods subsection from the original submission “Genetic variant clinical significance, functional category, minor allele frequency, and significant gene associations”. In this subsection all of the requested information regarding the providence of the data for the analyses done using Biomedical Data Commons was reported.

3. Study interpretation. The text, and many of the very complex figures do not lend themselves to a clear interpretation. There are many variants and loci associated with T1D – at the end there are some proposed mappings to putative regulatory areas of the HLA-DR locus. The “pipeline identified the IL2RA gene regulatory network with IL2RA as the top candidate within the locus by closeness centrality”. These are known loci and it is not fully clear that they were identified in analysis that used prior knowledge, or in naïve analyses that would have mapped and prioritized these loci. It is possible that the authors have explained the exact approach in the manuscript – but the truth is that the reader may have a lot of difficulty to understand the steps of analysis and the underlying assumptions and decisions.

We thank the reviewer for pointing out sections of the manuscript, which were insufficiently clear. To address this we added Figure 2, Supplementary Figure 1, and Supplmentary Figure 3, which all contain schematics that describe which data was used for which analyses and the data analysis workflow. In addition, Supplementary Table 3 of the  centrality score produced by the pipeline, which we used to prioritize genes and genetic variants within a locus. We also extensively edited the Results and Methods sections to provide further clarity into what was done, why these analyses were selected, and what the results mean. In particular, we greatly expanded the “Pipeline for prioritizing non-coding genetic variants” Methods subsection and added “Selection of the loci of interest” subsection to the Methods to clarify how the gene regulatory networks were produced and top genetic variants and gene candidates were identified within them. We used closeness centrality, a method that identifies highly connected nodes in a network, to prioritize regions of interest in gene regulatory networks. Closeness centrality is a method that has been used for years to identify critical nodes in social networks. We deploy it here to prioritize critical, highly connected genetic variants and genes in gene regulatory networks. As we show in the MHC Class II and IL2RA vignettes using closeness centrality identifies regions that are important for regulation of that locus as the top candidates identified in each have been previously validated in both mice and humans.

The study focus on B cells. However, comments such as the following creates doubts regarding the uniqueness of the B cell role given that many of the variants are described as shared with other cell lines (all in very generic terms): “Trio variants are largely cell-type specific to the input data used in the pipeline, but input data from GM12878, and 1° Naive T, Th17, and Treg cells mostly identified the same gene targets (Figure 2E)”

We thank the reviewer for pointing out that the reasoning behind the methods in this study were insufficiently clear. In response, we have added a paragraph to the introduction that introduces and explains the enhancer hypothesis. Enhancers are cis-regulatory regions that modulate gene expression. They are cell type specific. It’s known that distinct cell types have distinct enhancers to regulate their gene targets even if they’re the same. For example, in a given locus, there may be a B-lymphocyte-specific enhancer and a T-lymphocyte-specific enhancer each of which regulates the same gene target. Therefore, genetic variants that disrupt the function of B-lymphocyte-specific enhancers will lead to misregulation of gene targets in B-lymphocytes but not T-lymphocytes even if T-lymphocytes also express those genes. It is known that there are common genes implicated in B-lymphocytes and T-lymphocytes in the context of Type 1 Diabetes, but it’s less clear how cell type-specific regulation occurs. This provides an explanation for how gene regulation can be affected in only a subset of cells.

This has previously been demonstrated by Nott et al. 2019 in a Science paper in the context of Alzheimer’s Disease. The authors there used a similar approach to the one used here in which they used the three-dimensional chromatin and enhancer landscape to identify the gene targets of non-coding genetic variants in a cell-specific manner. They demonstrated that deletion of microglia-specific enhancers of BIN1 led to decreased expression of BIN1 in microglia and not astrocytes or neurons. Therefore, we would expect there to be distinct enhancers for B-lymphocytes and T-cells, which is what we observe in Figure 3E, left panel. We would also expect there to be a greater overlap in the enhancer gene targets in B-lymphocytes and T-cells, which is indeed what we reported in Figure 3E, right panel. These results are consistent with the enhancer hypothesis and previous literature.
