# Supplementary material for: Biomedical Data Commons (BMDC) prioritizes B-lymphocyte non-coding genetic variants in Type 1 Diabetes
Source: PLoS Comput Biol. 2021 Sep 20;17(9):e1009382. doi: 10.1371/journal.pcbi.1009382 (PMC8483327; doi:10.1371/journal.pcbi.1009382)
Supplement: Attachment — Submitted filename: Response to Reviewers Round 2.docx [file pcbi.1009382.s019.docx]

**Comments to the Authors:
Please note here if the review is uploaded as an attachment.**

Reviewer #1: The authors have adequately addressed our concerns. The manuscript is ready for publication.

We would like to thank reviewer 1 for their excellent feedback in the previous round of reviews and for recommending the paper for publication here.

Reviewer #2: no comments

We would like to thank reviewer 2 for their previous feedback that helped improve the manuscript and for recognizing that those concerns have been addressed.

Reviewer #3: Thanks for improving the readability of the manuscript.
One key comments, and a couple of minor remarks.
Two reviewers commented on the use of B cells instead of T cells. The authors provide an explanation in the anser to reviewers document that is perfectly acceptable: that this is the cell type that has extensive biological/genomic information. However, the paper provides a more novelty-based explanation implying that there is a biological interest. It would be preferable that the authors are as transparent with the readership and they were with the reviewers: the choice is a convenience decision, but may bring some biological insight on the role of B cells in diabetes, as well as indicating to what extent the work retrieved generalizable knowledge from B cells.

Thank you to reviewer 3 for recognizing how we addressed the reasoning behind the selection of B-lymphocytes for this study. We had previously added additional information in the Introduction and Discussion sections to further clarify why we decided to use B-lymphocytes as the main focus of the study. To further allay the concerns reviewer 3 has we added a sentence to the fifth paragraph of the Discussion that states that one of the reasons for selecting immortalized B-lymphocyte cell line as the focus of the study was that it was extensively characterized and has a wealth of publicly available epigenetic datasets. This reason was previously relayed to the reviewers only and is now included in the final manuscript.

Minor:
1. "The vast majority of output candidate variants are not evolutionarily conserved as defined by CADD score". The CADD score includes conservation, but it does not define conservation - other metrics go into CADD

Thank you to the reviewer for pointing out our minorly inaccurate wording. A huge component of CADD score is driven by evolutionary conservation, but other metrics are included. We therefore softened the language of the reviewer highlighted sentence in Results section and acknowledged that CADD scores are calculated using evolutionary conservation data alongside other metrics.

2. "Using the BMDC python API, we observed the majority of output candidate variants identified by the pipeline (S3 Supplementary Figure 3) to be common with a minor allele frequency (MAF) greater than 2%". By definition, if the input is GWAS information, it is expected that MAF will be 1-5%.

We would like to highlight that at this time we are referring to the variants identified by the pipeline. The input to the pipeline included all variants in linkage disequilibrium with GWAS identified significant variants. Most input variants into the pipeline as defined in Figure 2 were common (MAF > 0.05). Therefore, it is necessary to evaluate the minor allele frequency of the variants outputted by the pipeline.
